# Management of Renal Cell Carcinoma With Intra-atrial Tumour Thrombus: A Case Report

**DOI:** 10.7759/cureus.35380

**Published:** 2023-02-23

**Authors:** Ahmad S Alam, Kanica Yashi, Mostafa Elkhawaga

**Affiliations:** 1 General Surgery, John Hunter Hospital, Newcastle, AUS; 2 Internal Medicine, Bassett Healthcare, Cooperstown, USA; 3 Surgery, John Hunter Hospital, Newcastle, AUS

**Keywords:** cpb-cardiopulmonary bypass, venography, metastatic rcc, ivc tumor, renal cell carcinoma (rcc), right atrium tumor thrombus

## Abstract

Renal cell cancer (RCC) is at times associated with intravascular tumour thrombus (TT), which in rare cases can extend to the right atrium. The management of RCC with intravascular tumour thrombus is complex and requires a multidisciplinary approach involving urologists, vascular surgeons, and cardiologists. The pre-operative workup is extensive and includes imaging studies to determine the extent of the tumour thrombus and assess the patient's overall health status. Here, we present a case report detailing the operative and perioperative management of a patient presenting with renal cell cancer and intravascular TT.

## Introduction

In Australia, renal cell cancer is the seventh most diagnosed cancer, with an estimated 4552 new cases expected in 2020. The incidence rate of renal cancer in Australia is relatively low compared to other types of cancer, with around 15 new cases diagnosed per 100,000 people each year. The majority of renal cancers are diagnosed in people around the age of 70-74 years, and they are more common in men than in women [1]. Renal cell carcinoma is amongst the few tumours that show a disposition for an intravascular tumour thrombus (TT), with the incidence being reported at around 10% [2]. The tumour thrombus can extend all the way to the right atrium via the inferior vena cava (IVC), which has been shown to occur in around 1% of patients presenting with a TT [3].

## Case presentation

A 55-year-old female with a background history of asthma, hypertension and gastroesophageal reflux disease presented to the emergency department at a regional hospital with symptoms of frank haematuria with clots. She had complaints of nausea and lethargy with intermittent right upper quadrant pain for the past few months as well. Examination was unremarkable, with her observations being stable. Abdominal examination revealed a large, non-ballotable mass in the right upper quadrant that was minimally tender. The mass did not have regular borders and appeared to move with respiration. Her blood tests are detailed in Table 1. 

**Table 1:** Blood test results.

Blood chemistry	Result	Normal value
Urea	6.6 mmol/L	4-9 mmol/L
Creatinine	95 μmol/L	60-110 μmol/L
Estimated glomerular filtration rate	58 ml/min/1.73 m^2^	>60 ml/min/1.73 m^2^
White blood cell count	9.8 × 10^9^/L	4-11 × 10^9^/L
Haemoglobin	97 g/L	130-185 g/L

**Figure 1:**
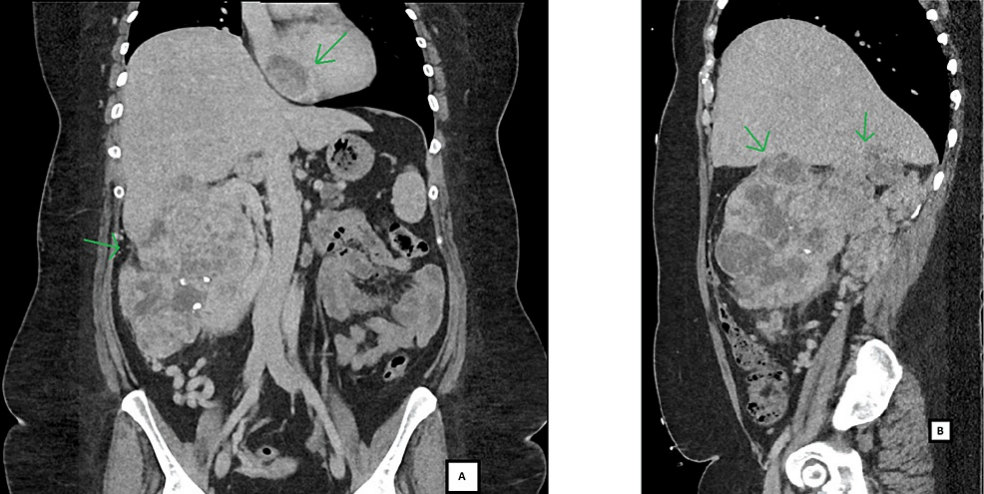
CT scan showing coronal (A) images of heterogenous mass with perinephric extension and right atrial lesion consistent with TT, and sagittal images (B) showing intrahepatic extension of mass. CT: computerised tomography, TT: tumour thrombus.

A computerised tomography (CT) scan of the abdomen revealed a large heterogeneous mass sized 19.1 × 9.7 × 19.6 cm with intrahepatic extension to segments 4b, 6 and 7 as well as perinephric extension (Figure 1A). The mass was also partially displacing the liver superiorly and the IVC, duodenum and pancreas to the left. A right atrial lesion measuring 3.8 × 6.1 × 3.8 cm, consistent with TT (Figure 1B), along with multiple retroperitoneal lymph nodes were observed as well. A transthoracic echocardiogram (TTE) done showed a heterogeneous mass of 6 × 3.2 cm extending from IVC and occupying most of the right atrial chamber and extending to the level of the tricuspid valve annulus.

**Figure 2:**
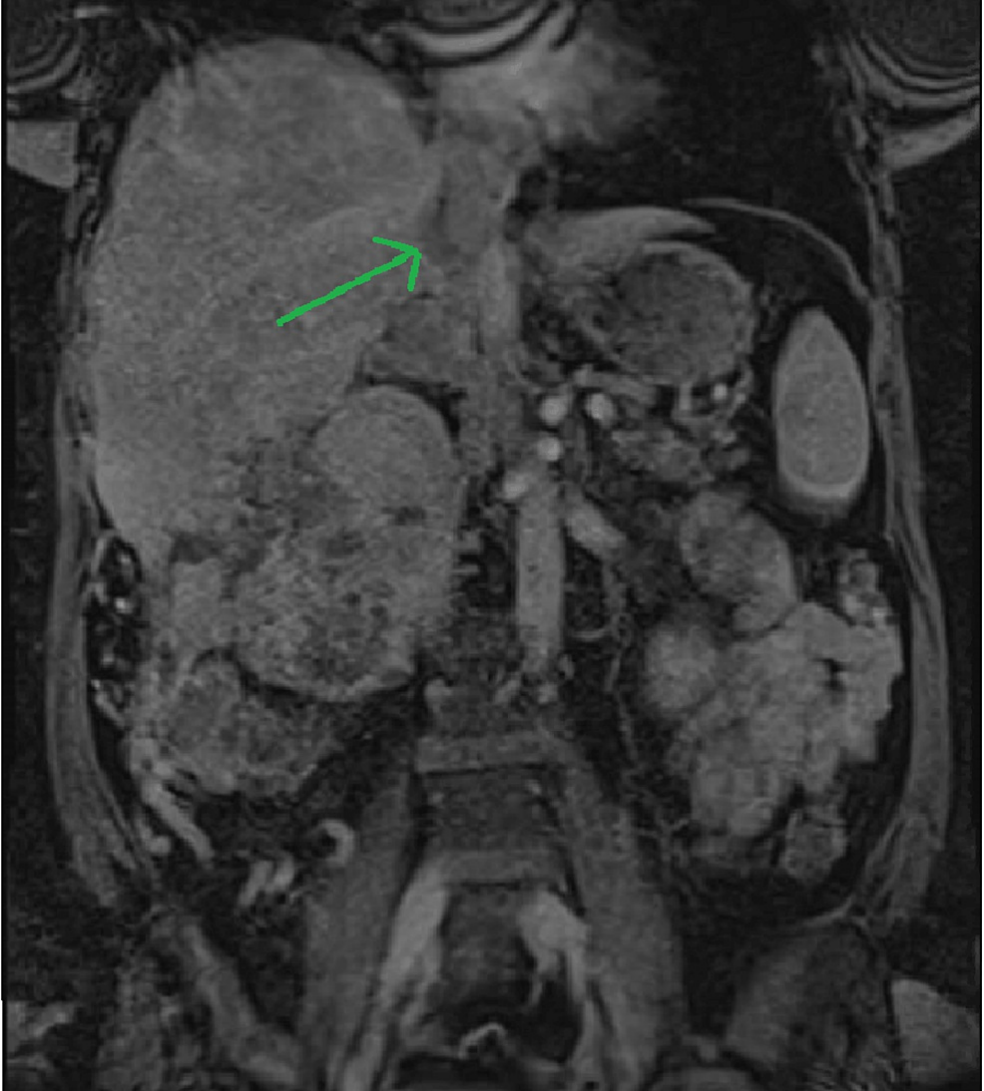
MRI showing vascular anatomy with TT in the IVC. TT: tumour thrombus, IVC: inferior vena cava, MRI: magnetic resonance imaging.

**Figure 3:**
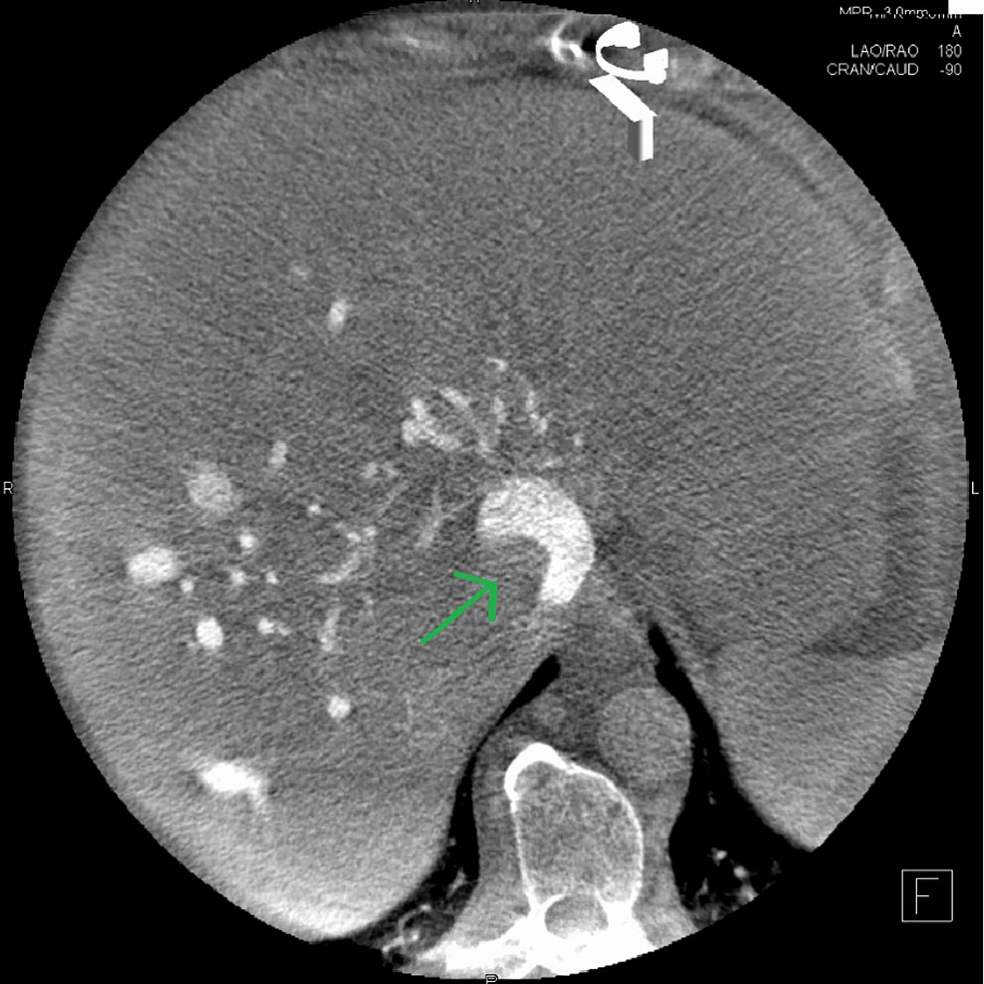
Venography demonstrating tumour adherent to cava wall.

The patient was admitted to our hospital under the urology and cardiothoracic team for observation and further investigations to help with surgical intervention. She underwent an magnetic resonance imaging (MRI) that revealed an invasion of the mass in the hepatic segments V and VI and clarified the vascular anatomy (Figure 2). The TT was abutting the tricuspid valve and bowing the interatrial septum into the left atrium. She underwent a diagnostic venogram to show tumour adherence to the caval wall at multiple places as well (Figure 3). She was discharged home with a plan to admit for surgery in four weeks.

On her next admission, she was taken to the operating room for an open right radical nephrectomy with IVC thrombectomy and retrieval of atrial thrombus under cardiac bypass, which has been described here.

Once the patient was intubated in a supine position, invasive monitoring was placed with an arterial line, a central line, and a transesophageal echocardiogram probe. A midline laparotomy was performed for mobilisation of the mass and exposure of the IVC, which was achieved after the falciform ligament, ascending colon and hepatic flexure were mobilised.

The cardiothoracic team then extended the midline laparotomy and entered the chest via a sternotomy. The renal vein and IVC were then isolated and separated from the perinephric tissue. Once the patient was heparinised, the aorta was clamped, and the patient’s core temperature was lowered to 20.5°C, after which cardioplegia was attained. The kidney was then mobilised and excised. The right atrial TT was retrieved via a right atriotomy. Inspection of the caudal IVC showed no obvious thrombus, but a further tumour was palpated at the level of the hepatic veins, which was mobilised digitally and retrieved via the right atrium. There was tumour invading the caval wall and hepatic vein as well, which could not be safely retrieved. There was significant bleeding from the liver bed after the mass was resected, and a hepatobiliary surgeon was consulted intraoperatively with the advice to use deep hepatic parenchymal sutures, packing and haemostatic adjuncts to manage the bleeding. The patient’s abdomen was packed, and an open abdominal vacuum-assisted closure (VAC) dressing was applied. She returned to the operation room two days later after an uneventful stay in the intensive care unit (ICU), where she remained intubated for an uncomplicated abdominal closure.

During her recovery on the ICU, she required continuous renal replacement therapy (CRRT) for oliguria and acute kidney injury (AKI), likely due to acute tubular necrosis (ATN) secondary to hypovolemia. She was gradually changed to intermittent haemodialysis (IHD) and started producing urine with an improvement of kidney function, after which her IHD was ceased 18 days postoperatively. She was also diagnosed with extensive bilateral deep vein thrombosis (DVTs), for which she was put on a heparin infusion and discharged home on a daily dose of clexane and a follow-up with haematology in six months. The patient also had to undergo review by speech pathology and ENT for a left vocal cord palsy secondary to intubation. She is due to follow-up with the ENT team in the clinics later next year and to continue with vocal function exercises to aid in swallowing.

Her histology was reviewed at our multidisciplinary team (MDT) meeting, which showed clear cell renal cell carcinoma, 220 mm, International Society of Urologic Pathologists (ISUP) Grade 2, pT3b, N0 and the TT showed clear cell renal carcinoma as well. She is due to get a repeat CT scan to assess the residual tumour and will then receive adjuvant radiotherapy as appropriate.

## Discussion

Intravenous neoplastic extension of TT has been classified according to the Mayo staging system into four levels namely, level 0 (thrombus extending to the renal vein); level I (thrombus extending into the IVC but no more than 2 cm above the renal vein); level II (thrombus extending into the IVC and 2 cm above the renal vein but not to the hepatic vein); level III (thrombus extending to the intrahepatic IVC); and level IV (thrombus extends above the diaphragm or into the right atrium) [4]. Although the process is not due to neoplastic infiltration of the vasculature but rather an intraluminal extension of the tumour, it is an indication of the aggressiveness of the neoplasm.

Intravascular TT, if completely resected along with a nephrectomy, has been shown to have a survival rate of around 47-62% at five years. This number drastically drops if the tumour has been incompletely resected or is associated with metastatic disease [5,6]. The level of the thrombus has not been demonstrated to have prognostic significance, except in cases where the tumour has encroached upon the atrium, which has been associated with a poorer prognosis [7].

The best imaging modality for diagnosing a TT has historically been venacavography, but this has recently fallen out of favour due to its inability to demonstrate caval wall invasion and the associated risk of kidney injury due to contrast [8]. Ultrasonography is usually used as the first line in diagnosing renal cell cancers with an intravascular thrombus, but it is not sensitive in detecting infrahepatic thrombi. Recent advances in CT scanning have increased the sensitivity of detecting renal cancers to 95%, but it struggles to precisely define the location of the thrombus due to the similar densities of blood and thrombus. The current gold standard used for surgical planning related to intravascular TT is magnetic resonance imaging (MRI), as it does not use contrast to delineate the thrombus and can provide multiplanar images [9]. Positron emission tomography (PET) has shown promising results; however, it is best utilised in complementing MRI scans and in patients with contrast allergies, renal failure, or occult malignancies [10]. Pre-operative and intraoperative transesophageal echocardiography are utilised in cases with a level III or level IV TT [7].

Management for renal cell carcinoma with a level IV thrombus involves complete resection of the parenchymal tumour and the TT while preventing tumour embolism and avoiding vital organ ischaemia. Most of the centres use cardiopulmonary bypass (CPB) and deep hypothermic circulatory arrest (DHCA) to treat tumours with intracardiac extension [11,12]. Other institutions have refrained from using DHCA due to its association with postoperative bleeding, coagulopathy, and prolonged bypass times, especially when aortic cross-clamping and the Pringle manoeuvre have been performed successfully.

At present, there is a lack of a universally recognised follow-up strategy and adjuvant therapy that is for patients with renal-cell carcinoma post-surgery that is supported by evidence. Current treatment protocols advocate for patient participation in a clinical trial or the implementation of active surveillance [13].

## Conclusions

Surgical intervention is the standard of care for renal cell cancer with intravascular tumour thrombus. Careful surgical planning and expertise are required to manage the tumour thrombus and its associated complications. This case report highlights the need for multidisciplinary management and close monitoring of postoperative complications in patients with complex renal cell cancer.
